# Cephalometric effects of Pushing Splints 3 compared with rapid maxillary expansion and facemask therapy in Class III malocclusion children: a randomized controlled trial

**DOI:** 10.1093/ejo/cjaa076

**Published:** 2020-12-12

**Authors:** Angela Galeotti, Stefano Martina, Valeria Viarani, Lorenzo Franchi, Roberto Rongo, Vincenzo D’Antò, Paola Festa

**Affiliations:** 1 Dentistry Unit, Department of Pediatric Surgery, Bambino Gesù Children’s Hospital, IRCCS, Rome, Italy; 2 Department of Medicine, Surgery and Dentistry ‘Scuola Medica Salernitana’, University of Salerno, Salerno, Italy; 3 Department of Experimental and Clinical Medicine, School of Dentistry, University of Florence, Florence, Italy; 4 School of Orthodontics, Department of Neurosciences, Reproductive Sciences and Oral Sciences, University of Naples “Federico II”, Naples, Italy

## Abstract

**Background:**

Pushing Splints 3 (PS3) device was recently introduced for the treatment of Class III malocclusion in children.

**Objectives:**

To assess the effect on the sagittal maxillary position (SNA, primary outcome) of PS3 therapy compared with rapid maxillary expansion and facemask therapy (RME/FM) and to compare skeletal and dento-alveolar effects in growing Class III patients.

**Trial design:**

This trial was a single-centre randomized controlled trial with two groups randomly allocated in a 1:1 ratio of equal size by sealed-envelope randomization, conducted at the Dentistry Unit of Bambino Gesù Children’s Hospital, IRCCS (Rome, Italy).

**Methods:**

A total of 48 patients with Class III malocclusion were included in the study and randomly allocated to the two groups: PS3 therapy and RME/FM therapy. Only the RME/FM group underwent palatal expansion, and both groups were instructed to wear the appliances 14 hours/day. Pre- (T0) and post-treatment (T1) cephalograms were taken. An independent sample *t*-test and regression analysis were used to analyse the data (*P* value <0.05). Researchers involved in statistics and tracings were blinded to the treatment allocation.

**Results:**

A total of 42 patients (21 for each group) completed the study. The maxillary sagittal position improved similarly in both groups (SNA = 0.4°; *P* = 0.547). A statistically significant decrease of SNPg angle (−1.6°; *P* < 0.001) and increase of ANPg angle (1.4°; *P* = 0.018) were found in the RME/FM group compared with PS3 group. CoGoMe angle significantly decreased in RME/FM group compared with PS3 group (−1.7°; *P* = 0.042). The regression analysis showed an association between SN/MP angle at T0 and the differences between T1 and T0 of SNPg (*B* = 0.13; *P* = 0.005) and SN/MP (*B* = −0.19; *P* = 0.034). Only three patients (PS3 = 2; RME/FM = 1) had breakages of the devices.

**Limitations:**

Results are limited to short-term effects.

**Conclusion:**

RME/FM therapy and PS3 are both effective therapies for the early correction of Class III malocclusion. The PS3 controlled better mandibular divergency reducing the clockwise rotation in patients with higher mandibular inclination.

**Registration:**

This study was not registered in a clinical trial registry.

## Introduction

Class III skeletal malocclusions may consist of maxillary retrognathism, mandibular prognathism, or a combination of both, along with several dento-alveolar and soft tissue compensations (1).

The prevalence of Class III malocclusions varies greatly among and within populations. According to a recent systematic review, in the permanent dentition, the global distributions of Class III malocclusion is 5.93% and in mixed dentition stage 3.98% (2) with a prevalence in Italy around the 5% (3). The complex aetiology of Class III involves genetic and environmental factors (4, 5).

The early approach remains a controversial topic in both academic and clinical fields (6). The central point of this lack of consensus is the inability to predict mandibular growth that is often unfavourable (7). However, early interceptive orthodontic treatment is suggested for several remarkable functional and aesthetic reasons although aware of the uncertainty of the long-term results (8). Early treatments mainly aim to influence the malocclusion development reducing the complexity of subsequent treatments (9).

Several treatment strategies have been reported in Class III malocclusion in children including Chincup (10), Frankel-3 (FR-3) (11), Reverse Twin-Block (12), Removable Mandibular Retractor appliance (13), Splints, Elastics and Chincup for Class III (SEC III) (14), Facial Mask (FM) (15), and Pushing Splints 3 (PS3) (16).

One of the most common orthopaedic treatment protocols for Class III malocclusion involves a combination of rapid maxillary expansion (RME) and facemask (FM) (15). This treatment protocol showed good short-term and long-term results (17). Regarding skeletal effects, it was evidenced that facemask induced forward displacement of maxilla, backward displacement of mandible associated to a clockwise rotation of the mandibular plane, and counter clockwise rotation of the maxillary plane (8, 18), for these reasons some authors suggested that facemask therapy is not indicated in hyperdivergent patients.

A new device called PS3 was recently introduced for treatment of Class III malocclusion in children (16, 19). Martina *et al.* (19) showed a significant improvement in sagittal skeletal relationships and no increase of vertical parameters with PS3 device. But this interesting study has some limitations: it is a retrospective study and the control group consisted of untreated subjects with skeletal Class III malocclusion from a database of longitudinal records collected in a different setting of the treated group.

The aim of this randomized clinical trial was to assess the effect on the sagittal maxillary position (SNA angle, primary outcome) of PS3 therapy compared with RME/FM, and to compare skeletal and dento-alveolar effects in growing Class III patients The null hypothesis is that there is no difference between the cephalometric changes on maxillary position induced by the PS3 compared with RME/FM in children.

## Materials and methods

### Trial design

**Figure 1.**
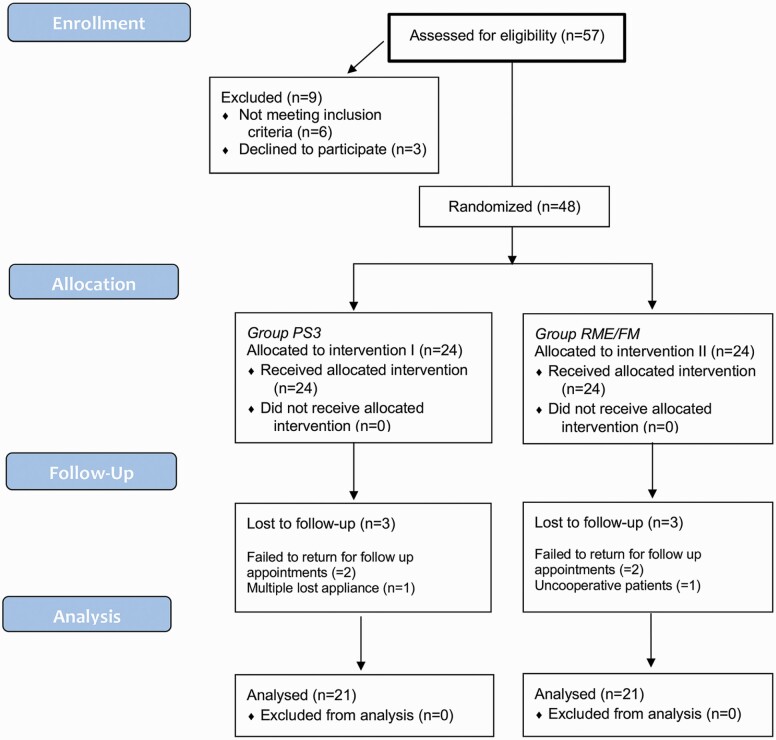
The CONSORT flow diagram.

This study was developed according to CONSORT (Consolidated Standards of Reporting Trials) statements. It consists in a single centre randomized controlled trial (RCT) with a 1:1 allocation ratio design, explained in the flowchart (Figure 1).

### Participants, eligibility criteria, and setting

This study was approved by the Ethics Committee of Bambino Gesù Children’s Hospital, IRCCS (Rome, Italy) (479_OPBG_2012). Written informed consent and assent forms were obtained from the parents and children to participate in the study.

The patients were recruited at the Dentistry Unit of Bambino Gesù Children’s Hospital, IRCCS (Rome, Italy) from February 2012 to June 2018.

The inclusion criteria for the study were:

- Caucasian ethnicity,
- deciduous, early or late mixed dentition,
- age between 4 and 10 years old,
- mesial step deciduous molar relationship or Class III permanent molar relationship in centric position,
- pre-treatment Wits appraisal of −2.0 mm or less.

Exclusion criteria were:

- no functional shift in occlusion,
- craniofacial anomalies,
- systemic disease affecting the normal growth patterns,
- clinically evident (more than 5%) facial and/or mandibular asymmetry,
- previous orthodontic treatment,
- impacted teeth,
- anomalies in dental morphology,
- periodontal disease,
- signs and symptoms of temporomandibular disorders.

Based on inclusion and exclusion criteria, a total of 48 patients were eligible for the study.

### Interventions and clinical procedures

#### Group PS3

**Figure 2.**
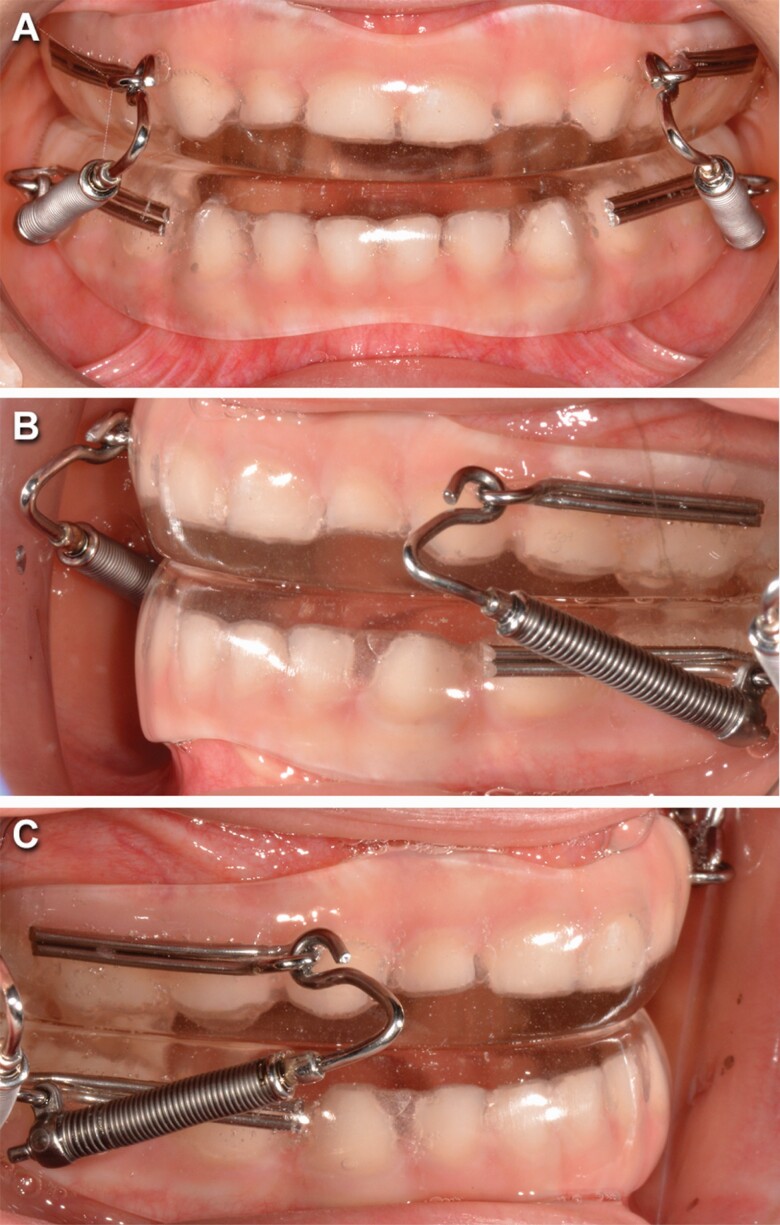
Pushing Splint 3 appliance in frontal intraoral (A), left-side intraoral (B), and right-side intraoral (C) views.

Impressions and a three layers intra-arches wax registration were taken to produce PS3 appliance. The PS3 appliance (Figure 2) consists of three components: two removable acrylic splints and one Forsus™ L-pin module per side. The two splints cover all the tooth crowns in both arches. Splints were empty on the vestibular surfaces of mandibular incisors if these teeth were too retroclined. Any pre-contact was removed. The Forsus™ modules were used to deliver a force of 200 g per side in a forward direction to the upper splint and in a backward direction to the lower splint. The Forsus™ L-pin produces a distalizing and intrusive vector on the lower molar and a mesializing and intrusive vector on the upper canine. The size of the Forsus™ L-pin was chosen evaluating the distance between the mesial of mandibular deciduous canine and the mesial of maxillary permanent first molar or the distal of maxillary deciduous second molar with a Forsus™ Device Gauge.

The coil springs were re-activated when necessary so that they were always be compressed. No expansion of the upper arch was performed. The patients were strongly motivated to wear the appliance for at least 14 hours a day and they were asked to record the daily wearing time in a diary.

#### Rapid maxillary expansion and facemask therapy

**Figure 3.**
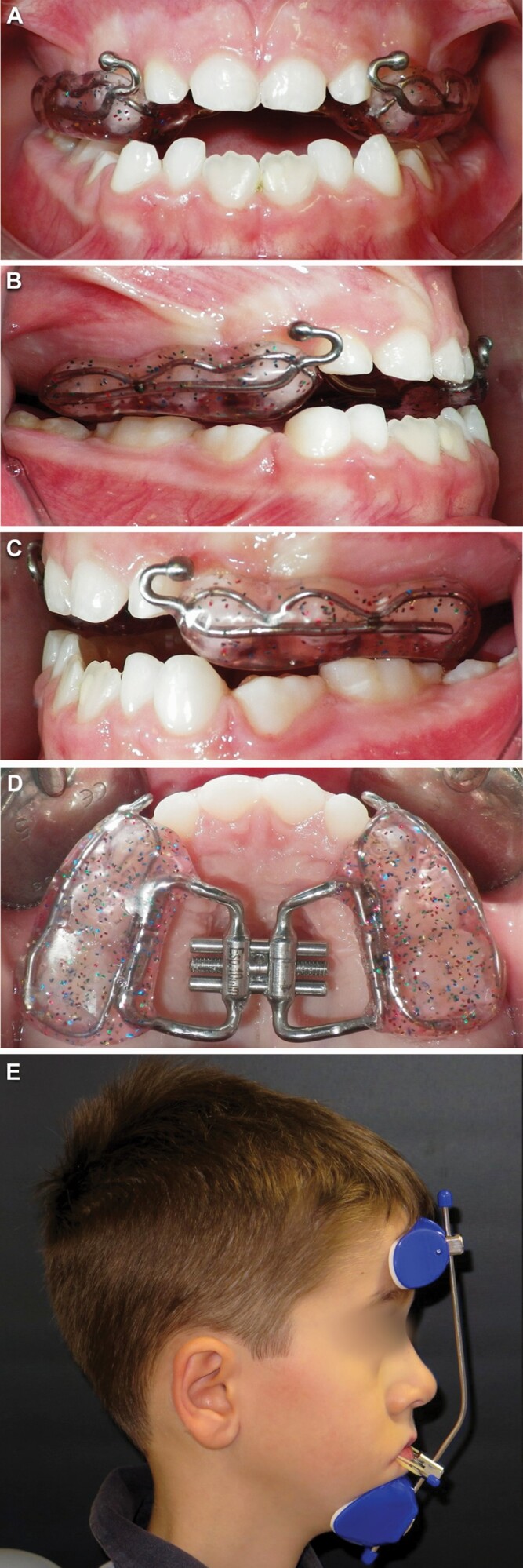
Bonded maxillary expander in intraoral upper occlusal view (A), frontal view (B), left-side view (C), and right-side view (D). Extraoral profile with Facemask (E).

Upper and lower arch impressions and intra-arches wax registration were taken in order to construct a bonded rapid maxillary expander with hooks for the FM (Figure 3). One-quarter activation of the screw was performed once a day until the palatal cusps of the upper molars approximated the buccal cusps of the mandibular molars even if no posterior crossbite existed. Then, the patient and his/her caregiver were instructed to wear the facemask using elastics connected downwards at 30° from the vestibular hooks on the RME to the facemask for 14 hours a day. Extra oral 3/8 8 oz elastics (one for each side) were prescribed for 10 days. When the patient showed good cooperation to the therapy, the force was increased to 350–400 g for each side using 5/16 14 oz elastics. The patients were strongly motivated to wear the appliance and they were asked to record the daily wearing time in a diary.

Both groups performed lateral cephalograms before (T0) and at the end of the treatment (T1). The active phase of the treatment ended when an overjet more than 2 mm was recorded and an overcorrection towards Class II molar relationship was achieved. After this active phase (on average 14 months), patients were asked to use the appliance only night-time as retention period (on average 4 months) and then the T1 lateral cephalogram was taken without the appliance.

### Outcomes

**Figure 4.**
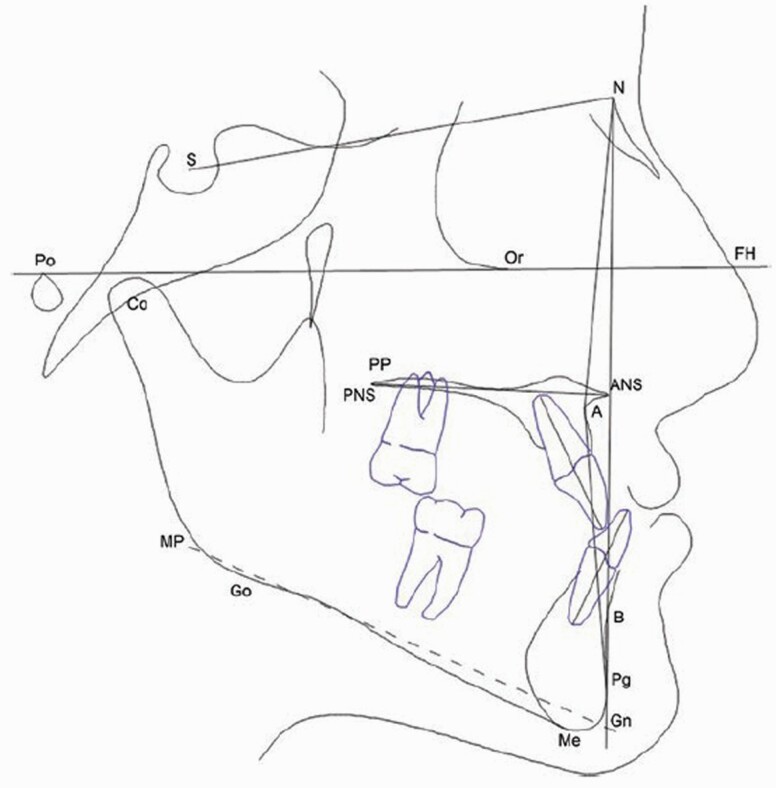
Cephalometric tracings.

Pre-treatment (T0) and post-treatment (T1) cephalometric tracings were analysed to compare skeletal and dental measurements. Cephalometric analyses (Figure 4) were performed using the Dolphin Imaging 11.0 software (Dolphin Imaging, Chatsworth, CA, USA). Each cephalogram was traced and 14 variables (5 linear and 9 angular) were measured. The measurements are described in Table 1. The primary outcome was to assess the effect on the sagittal maxillary position (SNA angle) of PS3 therapy compared with RME/FM therapy. The secondary outcome was to compare skeletal and dento-alveolar effects (secondary outcome) induced by the PS3 versus RME/FM therapy.

**Table 1.** Cephalometric landmarks, angles, and reference planes.

Measure	Definition
Sagittal skeletal	
SNA	The angle between the anterior cranial base and NA plane (°)
SNPg	The angle between the anterior cranial base and NPg plane (°)
ANPg	The angle between NA plane and NPg plane (°)
Wits appraisal	Distance between the two points of intersection of the two perpendicular lines from points A and B to the functional occlusal plane (mm)
Co-Gn	Mandibular base length from gonion to gnathion (mm)
Vertical skeletal	
SN/PP	Inclination of the palatal plane in relation to anterior cranial base (°)
SN/MP	Inclination of the mandibular plane GoGn in relation to anterior cranial base (°)
PP/MP	Inclination of the mandibular plane GoGn in relation to palatal plane (°)
CoGoMe	Angle between the condylar axis (Condylion–Gonion) and the mandibular base (Gonion–Menton)
Co-Go	Mandibular ramus height, distance between point Condylion and point Gonion (mm)
Interdental	
Overjet	The distance between maxillary incisor most labial and mandibular incisor edge parallel to occlusal plane (mm)
Overbite	The distance between maxillary incisor edge and mandibular incisor edge perpendicular to occlusal plane (mm)
U1/PP	Angle formed by intersection of maxillary incisor to palatal plane (°)
L1/MP	Angle formed by intersection of mandibular incisor to mandibular plane GoGn (°)

### Sample size calculation

The sample size was computed by using Gpower (Franz Faul, Universitat Kiel, Germany) considering *α* = 0.05, power = 0.90, an effect size of 1.05 considering an average difference between groups of 1.6° and a pooled standard deviation of 1.5° for the maxillary sagittal position (SNA angle) derived from a previous study (20). Hence, a sample size of at least 42 patients (21 patients for each group) was determined to be adequate comparing the groups with an unpaired *t*-test. To compensate for attrition, 48 patients were recruited.

### Randomization, allocation, concealment, and implementation

The patients were randomly allocated to the two groups. The randomization list was generated in randomization blocks of 10 with stratification according to gender. Hence, two randomization lists one for boys and one for girls was generated with the random generation function in Excel (Microsoft, Redmond, WA, USA). The numbers were sealed in opaque envelopes and the patients were randomly allocated into the two groups. One operator was responsible for opening the next envelope in sequence and implementing the randomization process.

### Blinding

During the study, it was impossible to blind the patients or the clinicians. However, the researcher measuring the lateral cephalograms and the researcher performing the statistical analysis were blinded to the treatment allocation. The cephalograms were labelled with numbers and randomly assessed by the researchers that did not know neither the timepoint nor the treatment.

### Method error

Measurement technical errors were calculated from 15 randomly selected patients at both T0 and T1. The same examiner digitized the same set of landmarks twice after a memory washout period of at least 6 weeks. The method error for all measurements was calculated using Dahlberg’s formula (21). Systematic differences between duplicated measurements were tested using a paired Student’s *t*-test with the type I error set at *P* < 0.05.

### Statistical analysis

Descriptive statistics included mean and standard deviation (SD) of cephalometric measurements at T0, T1, and for the T0–T1 interval. The normal distribution of the data was confirmed by the Shapiro–Wilk test. An independent samples *t*-test was used to compare the cephalometric variable at baseline (T0) and the changes during the T0–T1 interval between the two groups. A regression analysis was performed to evaluate the influence of the anterior cranial base-mandibular plane (SN-MP) angle on the changes between T1 and T0 of three sagittal and vertical skeletal variables (SNPg, ANPg, SN/MP). All statistical tests were two sided. *P* values less than 0.05 were considered significant. The dropouts were analysed evaluating their distribution between the two groups and the differences in the cephalometric values at T0 between the dropouts and the study participants. Standard statistical software package (SPSS version 22.0, SPSS, IBM, Armonk, NY, USA) was used for statistical analysis.

## Results

### Participants flow and recruitment

A total of 48 patients (24 males and 24 females; mean age ± SD = 7.1 ± 1.3 years) were randomized in a 1:1 ratio to either the PS3 group or the RME/FM group. Among them, six patients (three from each group, 3 males and 3 females; mean age ± SD = 7.7± 1.5 years) were lost to follow-up. The CONSORT flow chart of participants is shown in Figure 1.

### Numbers analysed

The method error ranged 0.04–0.5 mm for linear measurements, and from 0.04 to 0.7° for angular measurements. There was no systematic error for any of the 14 measurements (Student’s *t*-test; *P* > 0.05). The Shapiro–Wilk test showed that only four variables were not normally distributed (Supplementary Table 2).

The analysis of cephalometric variables at T0 showed no statistical differences between the dropouts and the children that completed the study (Supplementary Table 1). A total of 42 patients (21 in each group, 21 males and 21 females; mean age ± SD = 7.0 ± 1.2 years) were available for the statistical analysis.

### Baseline data

Analysis of the cephalometric measurements in RME/FM group and PS3 group at T0 showed no significant differences between the groups, indicating that the two groups presented similar clinical characteristics at baseline of the study (Table 2). There was also no significant difference between the two groups as regards distribution between the genders and average age: RME/FM group (7.2 ± 1.3 years) consisted of 11 females and 10 males while the PS3 group (7.0 ± 1.2 years) were 10 females and 11 males.

**Table 2.** Descriptive statistics and statistical comparisons of baseline characteristics.

	PS3		RME/FM		
	*n* = 21; *M* = 11; *F* = 10; Age = 7.2 ± 1.3 years		*n* = 21; *M* = 10; *F* = 11; Age = 7.0 ± 1.2 years		
Cephalometric measures	Mean	SD	Mean	SD	*P*
Sagittal skeletal					
SNA (°)	79.2	2.8	79.1	2.6	0.855
SNPg (°)	79.6	3.5	80.0	3.1	0.687
ANPg (°)	−0.3	2.5	−0.9	3.2	0.521
Wits (mm)	−5.1	1.7	−6.1	2.5	0.268*
Co-Gn (mm)	96.2	6.8	99.9	4.8	0.057
Vertical skeletal					
SN/PP (°)	7.2	2.8	7.2	3	0.975
SN/MP (°)	33	5.6	33.5	3.9	0.740
PP/MP (°)	28.5	5.9	28.8	4.3	0.851
CoGoMe (°)	126.1	5.6	127.1	4.4	0.519
Co-Go (mm)	42.5	3.6	43.8	3.0	0.080
Interdental					
Overjet (mm)	−1.1	1.6	−1.7	1.9	0.280
Overbite (mm)	1.6	1.1	1.1	1.3	0.080*
Maxillary dentoalveolar					
U1/PP (°)	106.4	9.4	105.9	7.7	0.876
Mandibular dentoalveolar					
L1/GoGn (°)	86.5	7.8	86.5	6.4	0.998

Significance level was set at *P* < 0.05. Data are reported as mean ± standard deviation.

*Mann–Whitney *U*-test.

The mean T0–T1 interval was 1.4 ± 0.4 years for PS3 group and 1.6 ± 0.4 years for RME/FM group and there was no statistical difference between groups.

Descriptive statistics and statistical comparisons for baseline characteristics and for the T1–T0 changes are, respectively, shown in Tables 2 and 3.

**Table 3.** Descriptive statistics and statistical comparisons of the T1–T0 changes.

	PS3			RME/FM						
	*n* = 21			*n* = 21						
Cephalometric measures	Mean	SD	95% CI	Mean	SD	95% CI	Mean difference	*P*	95% CI Lower limit	95% CI Upper limit
Sagittal skeletal										
SNA (°)	2.6	1.2	2.0;3.1	2.2	2.0	1.3;3.2	−0.4	0.547	−1.3	0.7
SNPg (°)	0.3	1.3	−0.2;0.9	−1.3	1.5	−2.0;0.7	−1.6	**0.000** ^*^	−2.5	−0.8
ANPg (°)	2.2	1.0	1.8;2.7	3.6	2.4	2.5;4.7	1.4	**0.018** ^*^	0.2	2.5
Wits (mm)	3.3	2.1	2.3;4.2	4.0	2.2	3.0;5.0	0.7	0.301	−0.6	2.1
Co-Gn (mm)	4.0	2.0	3.0;4.9	3.3	3.0	1.9;4.6	−0.7	0.394	−2.3	0.9
Vertical skeletal										
SN/PP (°)	−0.2	0.5	−0.4;0.0	0.0	1.6	−0.7;0.7	0.2	**0.036** ^*,**^	−0.5	0.9
SN/MP (°)	0.4	2.4	−0.6;1.5	1.1	2.9	−0.2;2.4	0.7	0.417	−1.0	2.3
PP/MP (°)	0.5	1.7	−0.2;1.3	1.0	2.7	−0.3;2.2	0.5	0.555	−1	1.8
CoGoMe (°)	0.4	2.7	−0.9;1.6	−1.3	2.3	−2.3;−0.2	−1.7	**0.042** ^*^	−3.2	−0.1
Co-Go (mm)	2.0	1.3	1.4;2.6	2.7	1.4	2.0;3.3	0.7	0.094	−0.1	1.6
Interdental										
Overjet (mm)	4.3	1.5	3.6;4.9	5.1	2.3	4.1;6.2	0.8	0.150	−0.3	2.1
Overbite	0.8	1.4	0.1;1.4	0.9	2.2	−0.1;1.9	0.1	0.473^**^	−1	1.3
Maxillary dentoalveolar										
U1/PP (°)	6.9	6.3	4.1;9.8	3.1	7.3	−0.3;6.4	−3.8	0.072	−8.1	0.4
Mandibular dentoalveolar										
L1/MP (°)	−1.8	5.5	−4.3;0.7	0.0	5.7	−2.6;2.6	1.8	0.315	−1.7	5.2

Significance level was set at *P* < 0.05. Data are reported as mean ± standard deviation (SD) and 95% confidence interval (95% CI). Bold type with asterisk (*): statistically significant.

^**^Mann–Whitney *U*-test.

### Outcomes and estimation

The results showed that the sagittal maxillary position advanced in both groups: SNA angle increase of 2.6° ± 1.2 in PS3 group and 2.2° ± 2.0 RME/MF with no statistical difference between groups (0.4°; *P* = 0.547).

The sagittal position of discrepancy improved in both groups: the ANPg angle increased by 2.2° ± 1.0 for PS3 protocol and by 3.6° ± 2.4 for RME/FM and the Wits appraisal increased by 3.3 mm ± 2.1 for PS3 and by 4.0 mm±2.2 for RME/FM.

A statistically significant decrease of SNPg angle (−1.6°) and increase of ANPg angle (1.4°) were found in the RME/FM group compared to PS3 group (*P* < 0.001 and *P* = 0.018 respectively).

As for the vertical skeletal variables, no statistically significant differences were found between groups except for CoGoMe angle that decreased significantly in RME/FM group compared to PS3 group (−1.7°; *P* = 0.042) and SN/PP angle (0.2°; *P* = 0.036).

Furthermore, no statistically significant differences were found between the groups for interdental and dentoalveolar measurements.

### Ancillary analysis

The regression analysis showed that the starting mandibular divergency influenced the changes in the vertical and sagittal mandibular cephalometric variables only in the PS3 group. Indeed, an association was found between SN/MP at T0 and the differences between T1 and T0 of SNPg (*B* = 0.13; *P* = 0.005) and SN/MP (*B* = −0.19; *P* = 0.034) (Table 4).

**Table 4.** Regression analysis assessing the influence of SN/MP (°) at T0 on the changes (T1–T0) of SNPg (°), ANPg (°), and SN/MP (°) for both Pushing Splint 3 (PS3) and facemask with rapid maxillary expansion (RME/FM).

	PS3			RME/FM		
	*n* = 21			*n* = 21		
Cephalometric measures	*B*	*P*	95% CI	*B*	*P*	95% CI
Sagittal skeletal						
SNPg (°)	0.13	**0.005** ^*^	0.05;0.22	0.04	0.646	−0.14;0.22
ANPg (°)	−0.07	0.086	−0.14;0.01	−0.18	0.201	−0.45;0.10
Vertical skeletal						
SN/MP (°)	−0.19	**0.034** ^*^	−0.37;−0.02	−0.17	0.312	−0.52;0.17

Significance level was set at *P* < 0.05. Data are reported as B (beta coefficient) and 95% confidence interval. Bold type with asterisk (*): statistically significant.

### Harms

In two patients of PS3 group, breakages in the portion between the Forsus™ L-pin and the lower splint occurred. Upper and lower impressions were taken again, and the broken portion was replaced by a dental technician.

In RME/FM group, a breakage of the vestibular hook occurred in one patient, the device was removed and the broken vestibular hook repaired by a dental technician. Some children encountered difficulties in maintaining good oral hygiene levels, they received further instructions on oral hygiene care at home and underwent oral hygiene treatment sessions.

The use of FM rarely created decubitus on the chin, the patients were suggested to suspend the use of the FM for some days and apply an ointment with Vitamin E until the skin appeared healed.

## Discussion

The aim of this randomized clinical trial was to compare the skeletal and dento-alveolar results of two different therapeutic approaches using the PS3 and bonded rapid maxillary expander associated to face mask (FM) in growing Class III patients.

### Generalizability

This study was carried out in a clinical environment similar to many of the contemporary orthodontic practice. The results of the present study can be generalized for patient groups with similar mean age, inclusion/exclusion criteria, and treatment protocol.

### Interpretation

In this study, both the appliances improved the relations between the maxillary and mandibular position, in growing children.

Westwood *et al.* in a study on the effects of conventional RME/FM therapy for Class III malocclusion found similar results (22). The efficacy of RME/FM protocol was deeply investigated and confirmed by an interesting systematic review and meta-analysis (18) while the skeletal effects of PS3 for correcting Class III malocclusion in children was previously reported only in a case report (16) and in a retrospective study that compared the study group to a control group selected from a database of subjects with untreated Class III malocclusion (19).

The analysis of the dropout showed an equal distribution (three vs three) between the two groups, this might suggest that the two appliances affect similarly the patient’s willingness to withdraw from the study. Moreover, the assessment of the cephalometric variables showed no differences between the children included in the study and the dropouts.

Both protocols were able to induce a similar favourable maxillary advancement as shown by the increase in SNA angle. These results are consistent with the outcomes of several studies that evaluated the effect of RME/FM therapy in growing patients (23, 24).

The mandibular position evaluated with SNPg and ANPg angles showed a statistically significant reduction with RME/FM protocol compared to the PS3 group. However, a similar reduction of SNB angle was evidenced by a meta-analysis regarding maxillary protraction by FM (25). Hence, this study showed a better efficacy of FM in controlling the sagittal position of the mandible compared with PS3 and suggested that this therapy might be preferred in Class III malocclusion children with a major component of mandibular prognathism.

Small differences in vertical variables were recorded between the two treatments protocol in this study. In the RME/FM group, the post-treatment amount of clockwise rotation of the mandibular plane was very limited similar to previous findings (26). We suppose the use of a correct downward inclination of extraoral elastics at 30° to the occlusal plane limited the negative side effects of RME/FM treatment in terms of mandibular clockwise rotation as previously suggested (27).

Furthermore, CoGoMe angle exhibited a statistically significant decrease in RME/FM group compared to the PS3 group. We might relate these data to different biomechanics: the force applied to the chin in a cranial and posterior direction might re-direct the mandibular rotation pattern such as suggested by Franchi *et al.* (28). This growth modification (anterior morphogenetic rotation) has been advocated as a favourable mechanism to dissipate mandibular growth excess that can be a favourable aspect in Class III patients (29). However, also the PS3 group showed a good control of the mandibular divergency evaluated on anterior cranial base.

In a recent study, Salazar *et al.* (30) evaluated the effect of maxillary protraction with facemask therapy on mandibular rotation and evidenced most participants maintained their initial vertical growth pattern. In particular, they reported that four patients out of 29 with initial clockwise rotation shifted towards a reduction of the anterior cranial base-mandibular plane (SN-MP) angle after therapy. In our study, a regression analysis was performed to evaluate how the mandibular divergency influences the sagittal and vertical skeletal changes due to the two appliances. Interestingly, the mandibular divergency was associated to skeletal changes only for the PS3 appliance. Indeed, the regression analysis showed that for higher values of anterior cranial base-mandibular plane (SN-MP) angle at T0, the mandibular advancement between T1 and T0 increases while the clockwise rotation of the mandible decreases. These data confirm that the use of PS3 could be more indicated in hyperdivergent patients.

A significant overjet correction was obtained with both treatments RME/FM and PS3, while overbite did not change significantly after therapy. Results provided by Rongo *et al.* in a meta-analysis carried out on this topic are quite consistent (8). The effect of maxillary protraction through FM did not cause significant protraction of the maxillary incisors or retraction of the mandibular incisors and these findings are similar to those reported by Cozza *et al.* (31). Dento-alveolar changes in the PS3 group are like those reported by Martina *et al*. (19). Although it was not statistically significant, the comparison between groups highlights a greater protrusion of the upper incisors and retrusion of the lower incisors in the PS3 group compared with the FM group. It might depend on the fact that PS3 covered upper and lower incisors whose inclinations were affected by sagittal forces.

On the other hand, the PS3 is a removable device and it might be preferred to the bonded expander in patients when the use of fixed appliances are not preferable for tolerance problems, poor oral hygiene, or tooth eruption.

### Limitations

The results of this study were limited to a short-term observation period immediately and, therefore, further studies are needed to evaluate the long-term effect of PS3 therapy. Furthermore, it was not possible to blind the clinicians and the patients and the study was not registered in a clinical trial registry.

## Conclusions

The results of the present study indicate that RME/FM therapy as well as PS3 are effective therapies for the early correction of Class III malocclusion. However, the FM therapy allows obtaining more favourable effects in the control of mandibular position and a greater mandibular anterior morphogenetic rotation compared with PS3, so it might be preferred in Class III patients with a major component of mandibular protrusion.

On the other hand, PS3 seems to have a better control of the mandibular divergency reducing the clockwise rotation in patients with higher mandibular inclination on the anterior cranial base.

## Supplementary Material

cjaa076_suppl_Supplementary_Table_1

cjaa076_suppl_Supplementary_Table_2

## Data Availability

The data that support the findings of this study are available on request from the corresponding author [AG]. The data are not publicly available for containing information that could compromise research participant privacy/consent.
