# Supplementary material for: Cephalometric effects of Pushing Splints 3 compared with rapid maxillary expansion and facemask therapy in Class III malocclusion children: a randomized controlled trial
Source: Eur J Orthod. 2020 Dec 12;43(3):274–82. doi: 10.1093/ejo/cjaa076 (PMC8186836; doi:10.1093/ejo/cjaa076)
Supplement: cjaa076_suppl_Supplementary_Table_1 [file cjaa076_suppl_supplementary_table_1.docx]

|  | **Participants** | | | **Dropouts** | | |  |
| --- | --- | --- | --- | --- | --- | --- | --- |
| Cephalometric measures | n=42 |  |  | n=6 |  |  |  |
| *Sagittal Skeletal* | Mean | SD | 95% CI | Mean | SD | 95% CI | P |
| SNA (°) | 79.2 | 2.6 | 78.3;80 | 78.7 | 3.7 | 74.9;82.6 | 0.781 |
| SNPg (°) | 79.8 | 3.2 | 78.8;80.8 | 79.7 | 3.3 | 76.3;83.2 | 0.967 |
| ANPg (°) | -0.6 | 2.8 | -1.5;0.3 | -1 | 0.8 | -1.9;-0.1 | 0.487 |
| Wits (mm) | -5.7 | 2.1 | -6.3;-5 | -3.5 | 2.4 | -6;-1 | 0.078 |
| Co-Gn (mm) | 97.5 | 6.4 | 95.5;99.5 | 100.5 | 2.9 | 97.4;103.6 | 0.073 |
| *Vertical Skeletal* |  | | | | | | |
| SN/PP (°) | 7.2 | 2.8 | 6.3;8.1 | 2.4 | 4.8 | -2.6;7.4 | 0.056 |
| SN/GoGn (°) | 33.2 | 4.8 | 31.8;34.8 | 32.2 | 4.0 | 28;36.5 | 0.587 |
| ANS-PNS/GoGn (°) | 28.6 | 5.1 | 27.1;30.2 | 27.3 | 2.5 | 24.6;29.9 | 0.319 |
| CoGoMe (°) | 126.6 | 4.9 | 125.1;128.1 | 123.5 | 3.6 | 119.7;127.3 | 0.098 |
| Co-Go (mm) | 42.9 | 3.4 | 41.9;44 | 45.7 | 3.1 | 42.5;49 | 0.083 |
| *Interdental* |  | | | | | | |
| Overjet (mm) | -1.4 | 1.8 | -2;-0.9 | -1.7 | 3.3 | -5.2;1.7 | 0.834 |
| Overbite | 1.3 | 1.2 | 0.9;1.7 | 2.4 | 1.7 | 0.6;4.3 | 0.183 |
| *Maxillary dentoalveolar* |  | | | | | | |
| U1/PP (°) | 106.1 | 8.5 | 103.5;108.8 | 103.1 | 7.6 | 95.2;111.1 | 0.396 |
| *Mandibular dentoalveolar* |  | | | | | | |
| L1/GoGn (°) | 86.5 | 7.0 | 84.3;88.6 | 88.9 | 3.2 | 85.5;92.3 | 0.171 |

Supplementary table 1
