# Supplementary material for: Cephalometric effects of Pushing Splints 3 compared with rapid maxillary expansion and facemask therapy in Class III malocclusion children: a randomized controlled trial
Source: Eur J Orthod. 2020 Dec 12;43(3):274–82. doi: 10.1093/ejo/cjaa076 (PMC8186836; doi:10.1093/ejo/cjaa076)
Supplement: cjaa076_suppl_Supplementary_Table_2 [file cjaa076_suppl_supplementary_table_2.docx]

| Cephalometric measures | T0 | | T1-T0 | |  |
| --- | --- | --- | --- | --- | --- |
| *Sagittal Skeletal* | |  | |  | |
| SNA (°) | | 0.324 | | 0.798 | |
| SNPg (°) | | 0.671 | | 0.307 | |
| ANPg (°) | | 0.861 | | 0.050 | |
| Wits (mm) | | **0.030*** | | 0.983 | |
| Co-Gn (mm) | | 0.192 | | 0.668 | |
| *Vertical Skeletal* | |  | |  | |
| SN/PP (°) | | 0.211 | | **<0.001*** | |
| SN/MP (°) | | 0.877 | | 0.605 | |
| PP/MP (°) | | 0.983 | | 0.111 | |
| CoGoMe (°) | | 0.780 | | 0.829 | |
| Co-Go (mm) | | 0.065 | | 0.245 | |
| *Interdental* | |  | |  | |
| Overjet (mm) | | 0.134 | | 0.547 | |
| Overbite (mm) | | **0.012*** | | **0.002*** | |
| *Maxillary dentoalveolar* | |  | |  | |
| U1/PP (°) | | 0.474 | | 0.679 | |
| *Mandibular dentoalveolar* |  | |  | |  |
| L1/GoGn (°) | 0.732 | | 0.123 | |  |

Supplementary table 2
